# Molecular evidence of sporadic *Coxiella burnetii* excretion in sheep milk, central Portugal

**DOI:** 10.1007/s11259-024-10389-x

**Published:** 2024-04-24

**Authors:** Humberto Pires, Sérgio Santos-Silva, Andreia V.S. Cruz, Luís Cardoso, Ana Patrícia Lopes, Maria A. Pereira, Carmen Nóbrega, Ana Cristina Mega, Carla Santos, Rita Cruz, Fernando Esteves, Helena Vala, Ana Cristina Matos, Patrícia F. Barradas, Ana Cláudia Coelho, João R. Mesquita

**Affiliations:** 1 Polytechnic Institute of Castelo Branco, Castelo Branco, 6001-909 Portugal; 2 ICBAS – School of Medicine and Biomedical Sciences, Porto University, Porto, 4050-313 Portugal; 3 Animal and Veterinary Research Centre (CECAV), Department of Veterinary Sciences, University of Trás-os-Montes e Alto Douro (UTAD), Vila Real, 5000-801 Portugal; 4 Associate Laboratory for Animal and Veterinary Sciences (AL4AnimalS), Vila Real, 5000-801 Portugal; 5 Instituto Politécnico de Viseu, Escola Superior Agrária de Viseu, Campus Politécnico, Viseu, 3504-510 Portugal; 6 Global Health and Tropical Medicine, Associate Laboratory in Translation and Innovation Towards Global Health, GHTM, LA-REAL, Instituto de Higiene e Medicina Tropical, IHMT, Universidade NOVA de Lisboa, UNL, Rua da Junqueira 100, Lisboa, 1349-008 Portugal; 7 CERNAS-Research Centre for Natural Resources, Environment and Society, ESAV, Instituto Politécnico de Viseu, Viseu, 3500-606 Portugal; 8 Centre for the Research and Technology of Agro-Environmental and Biological Sciences (CITAB), University of Trás-os-Montes e Alto Douro, Vila Real, 5001-801 Portugal; 9 Epidemiology Research Unit (EPIUnit), Instituto de Saúde Pública da Universidade do Porto, Porto, 4050-091 Portugal; 10 Laboratório para a Investigação Integrativa e Translacional em Saúde Populacional (ITR), Porto, 4050- 600 Portugal; 11 Research Center for Natural Resources, Environment and Society, Polytechnic Institute of Castelo Branco, Castelo Branco, 6001-909 Portugal; 12 Quality of Life in the Rural World (Q-RURAL), Polytechnic Institute of Castelo Branco, Castelo Branco, 6001- 909 Portugal; 13 Department of Sciences, CESPU, CRL, University Institute of Health Sciences (IUCS), Gandra, Portugal

**Keywords:** Bulk milk, ***Coxiella burnetii***, One health, Portugal, Sheep

## Abstract

*Coxiella burnetii* is the etiologic agent of Q fever, a worldwide zoonosis. Cattle, sheep and goats are considered the main reservoirs of the disease. Transmission to humans occurs mainly through the inhalation of infectious aerosols from milk, faeces, urine, and birth products from infected ruminants. In this study, a 2-year longitudinal approach was performed to ascertain the excretion of *C. burnetii* in bulk tank milk samples of sheep from a mountain plateau in central Portugal, with sampling conducted during the years 2015 and 2016. From a total of 156 bulk tank milk samples tested by qPCR, only one showed to be positive for *C. burnetii* (1.28% [95%CI: 0.03–6.94]), from 2015, the first year of collection. Bidirectional sequencing and phylogenetic analysis of *IS1111* transposase partial region confirmed the presence of *C. burnetii* DNA. The presence of *C. burnetii* in raw milk samples highlights the necessity for additional research to determine if raw milk is a potential source for human infection. Animal health surveillance and prevention measures against this zoonotic disease should be considered.

## Introduction

*Coxiella burnetii* is the etiologic agent of Q fever, a widespread zoonosis (Genova-Kalou et al. 2021) and is considered a potential bioterrorism agent and classified as a Group B biological agent by the Centre for Disease Control and Prevention (CDC) (http://emergency.cdc.gov/bioterrorism/overview.asp 2007; Sahu et al. 2020).

*Coxiella burnetii* is a small Gram-negative coccobacillus (family Coxiellaceae, order Legionellales), being an obligate intracellular bacterium that replicates in eukaryotic cells. This bacterium occurs in two forms: the large-cell variant (LCV), an exponentially replicating form, and the small-cell variant (SCV), a stationary nonreplicating form, stable in the environment and highly resistant (Eldin et al. 2017).

This bacterium can infect a wide range of hosts, including humans, ruminants, birds, reptiles, fish, and ticks (Cutler et al. 2007). The main reservoirs and the most common sources of human infection are cattle, sheep, and goats (Angelakis and Raoult 2010). The clinical presentation depends on the virulence of the infecting strain, the route of infection and the risk factors of the host (Eldin et al. 2017). The infection in humans can range from asymptomatic or subtly symptomatic, with symptoms mistaken for flu-like illnesses, to chronic or even fatal, usually through endocarditis (Cutler et al. 2007; Angelakis and Raoult 2010). In contrast, *C. burnetii* infection in animals is typically subclinical, making the term coxiellosis more appropriate (Angelakis and Raoult 2010). However, abortions and stillbirths can occur in infected sheep and goats, mainly during late pregnancy (van den Brom et al. 2015).

From 2007 to 2010, the Netherlands experienced the largest outbreak of Q fever ever reported, with over 4000 registered human cases and culling of thousands of small ruminants (Delsing et al. 2010). This outbreak prompted a reassessment of the risks for pregnant women. However, no evidence was found for adverse effects on pregnancy outcomes, among pregnant women with asymptomatic infections in early pregnancy which might be attributed to a possible difference in pathogenicity of different strains. The Netherlands outbreak underscored the unpredictability of the sudden emergence and spread of *C. burnetii* (Eldin et al. 2017). In Portugal, Q fever is considered an endemic disease in humans, and has been an obligatory notifiable disease since 1999 (Palmela et al. 2012). Studies on ruminants have also shown not only a high seroprevalence (Cruz et al. 2018a), but also high levels of *C. burnetii* detection (Clemente et al. 2009; Cruz et al. 2018b). However, disease in humans is considered to remain undiagnosed and underreported in the country (Palmela et al. 2012).

Transmission to humans may be due to inhalation of infectious aerosols from milk, faeces, urine, and birth products from infected ruminants (Cutler et al. 2007). Moreover, the risk of infection in humans is predominantly determined by the shedding of *C. burnetii* during lambing. Despite conventional transmission routes, consuming raw milk and dairy products originating from contaminated raw milk has also been increasingly considered (Angelakis and Raoult 2010; Eldin et al. 2017; Pexara et al. 2018). For example, recent reports have identified these bacteria in raw cow’s milk intended for human consumption (de Souza Ribeiro Mioni et al. 2019) and another study detected viable *C. burnetii* in sheep hard cheeses made with unpasteurized milk (Barandika et al. 2019). Furthermore, studies have also reported the shedding of these bacteria in milk from ruminants (Mobarez et al. 2021; Kalaitzakis et al. 2021).

Considering the lack of knowledge regarding the epidemiology of *C. burnetii* in Portugal, this study aimed to perform a 2-year longitudinal approach to ascertain the excretion of *C. burnetii* in bulk tank milk (BTM) samples of sheep from a mountain plateau in central Portugal.

## Materials and methods

### Bulk tank milk collection

**Fig. 1:**
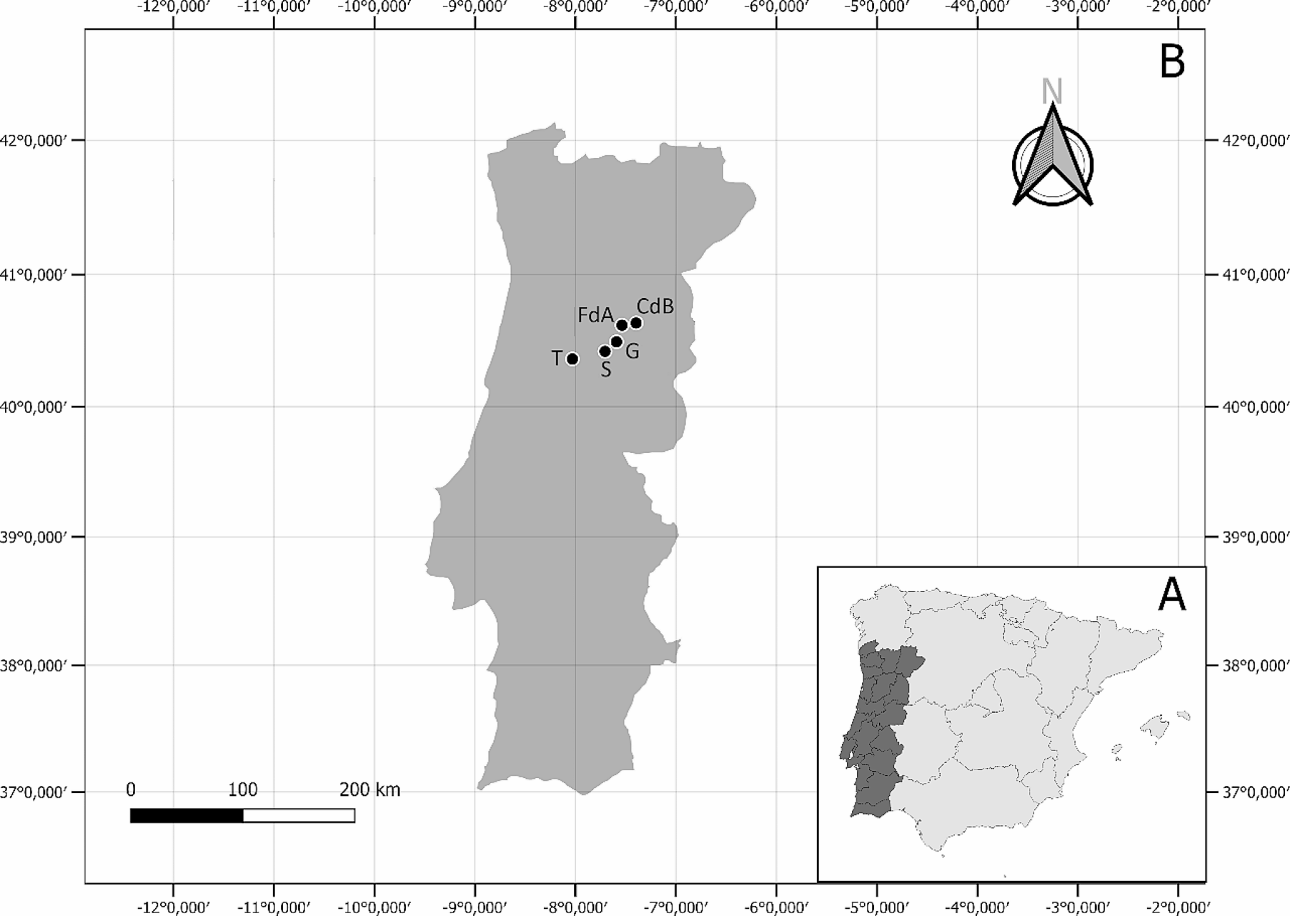
Geographic distribution of the sheep milk farms sampled in the Centre region of Portugal. A: Iberian Peninsula; B: Portugal; CdB: Celorico da Beira; FdA: Fornos de Algodres; G: Gouveia; S: Seia; T: Tábua. Figure elaborated using QGIS 3.34.3

For the *C. burnetii* screening, samples from a previous study on Schmallenberg virus (Esteves et al. 2019) were used. For this study all officially registered sheep flocks from Portugal’s Centre region, 180 in total (http://www.ancose.com), were invited to contribute, involving the collection of BTM at two points in time (January/February 2015 and January/February 2016). Out of these, 78 sheep dairy farms located across 46 parishes within five municipalities of Portugal’s Centre region (namely Celorico da Beira, Fornos de Algodres, Gouveia, Seia, and Tábua) (Fig. 1) agreed to participate, marking a participation rate of 43.3%. A 2 mL sample of bulk milk was collected into sterile plastic tube from each farm during both (2015/2016) collection periods, summing up to 156 BTM samples. These samples were then promptly transported to a laboratory and maintained at 4 °C.

### DNA extraction

DNA was extracted using the DNeasy Blood and Tissue Kit (Qiagen, Hilden, Germany) according to the manufacturer’s instructions and using the QIAcube® automated platform (Qiagen). Before extraction, milk samples were centrifuged at 1000 × *g* for 10 min as previously described (Blackwell et al. 1982). Afterwards, the supernatant was rejected and the cell fraction resuspended in PBS (pH 7.4) and centrifuged for 15 min at 1700 × *g* (Renshaw et al. 2000), discarding the supernatant afterwards. The pellet was resuspended in PBS (pH 7.4) and centrifuged for 15 min at 1700 × *g*, until residual cream was removed (Basanisi et al. 2022). Final pellet was resuspended and subjected to extraction in 180 µL of buffer ATL. DNA extracts were stored at − 20 °C until analysis.

### Molecular analysis

For the initial screening, a SyBr green real-time PCR (qPCR) test was used (Vaidya et al. 2010; Capuano et al. 2012). This qPCR targets the *IS1111* transposase which is present in a variable number of copies in different isolates of *C. burnetii* (Klee et al. 2006),with the Nine Mile reference strain presenting 20 copies (Seshadri et al. 2003). The qPCR reactions were conducted in a total volume of 25 µL using the Xpert Fast SYBR uni (GRiSP®, Porto, Portugal), according to the manufacturers’ instruction, and the primer pair Trans 3 F and Trans 4 R.The thermal profile of qPCR assays used was as follows: 95 °C for 3 min followed by 50 cycles of 95 °C for 5 s and an annealing of 61 °C for 30 s with acquisition of fluorescent data. After the PCR cycles, a melting curve (TM) was generated (30 s at 61 °C, 30 s at 95 °C) to discriminate between the specific amplicons and non-specific amplification products. The TM value was defined as the peak of the curve. qPCR reactions were run on a CFX Connect Real-Time PCR Detection System (Bio-Rad, Hercules, CA, USA) and data were analyzed using the CFX Maestro 1.0 Software version 4.0.2325.0418 (Bio-Rad, Hercules, CA, USA).

For confirmation and genetic characterization of positive samples, an endpoint PCR was performed. The endpoint PCR reactions were conducted in a total volume of 25 µL using the Xpert Fast Hotstart Mastermix (GRiSP®, Porto, Portugal), according to the manufacturer’s instructions, and the same primers (243 bp) as the qPCR (Capuano et al. 2012).The thermal profile of endpoint PCR assays used was as follows: 95 °C for 3 min followed by 40 cycles of 95 °C for 15 s, annealing of 61 °C for 15 s, extension of 72 °C for 2 s, and a final extension of 72 °C for 10 min with a hold of 12 °C. All endpoint PCR reactions were performed on a T100 thermocycler (Bio-Rad, Hercules, CA, USA). After PCR amplification, the DNA fragments were separated and visualized through electrophoresis on 1.5% agarose gels stained with Xpert Green Safe DNA gel dye (GriSP®, Porto, Portugal). Electrophoresis was carried out at 120 V for 25 min. To visualize the results, UV light was used for irradiation of the agarose gels.

### Sequencing and phylogenetic analysis

Amplicons showing presumptively positive sizes were purified using the GRS PCR and Gel Band Purification Kit (GriSP®, Porto, Portugal). Following purification, bidirectional sequencing was performed and edited using the BioEdit Sequence Alignment Editor v7.1.9 software package, version 2.1. Resulting consensus sequences were compared to those present in the NCBI (GenBank) nucleotide database, accessed on 25 January 2024.

MEGA version X software was employed for additional analysis and interpretation of the sequences (Kumar et al. 2018). The Jukes-Cantor model was used to estimate the ML bootstrap values using 1000 replicates. Models function on MEGA version X was used to opt for the model with the smallest Bayesian information criterion (BIC) score (Zhang et al. 2018). The sequence obtained in this study was deposited in GenBank with accession number OL310491.

## Results

From the initial qPCR screening of the 78 BTM samples from 2015, only one (1.28%) showed a melting curve with a TM compatible with the expected 88.1 ± 0.3 °C. This sample was collected from a sheep farm in Gouveia. From the qPCR screening of the 78 BTM samples from 2016 none showed to be positive.

### *Coxiella burnetii*

**Fig. 2:**
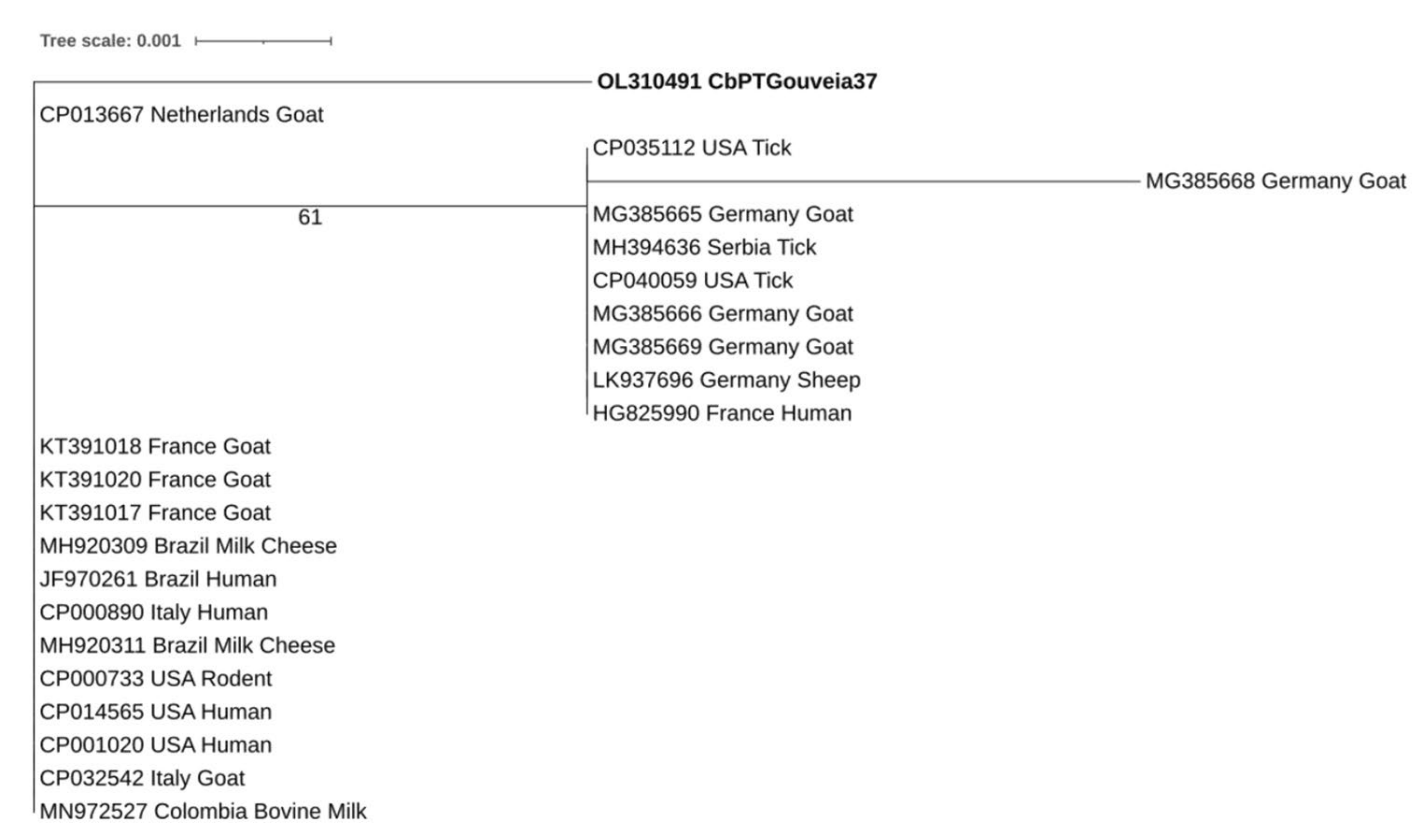
Phylogenetic analysis of *Coxiella burnetii* sequence found in a bulk tank milk sample from Portugal. *Coxiella burnetii* found in this study (accession no: OL310491) is highlighted in bold. The tree was inferred using the MEGA X software and the Interactive Tree of Life (iTOL) based on 23 nucleotides *Coxiella burnetii* sequences, including the one detected in this study

Bidirectional sequencing followed by nBLAST analysis confirmed the presence of *C. burnetii* DNA showing highest hits (100%) with several sequences including *C. burnetii IS1111* transposase partial sequences found in brown dog tick (*Rhipicephalus sanguineus;* KT867378), dog (*Canis lupus familiaris;* KT867377) and goat (*Capra hircus*; CP013667). Phylogenetic analysis was performed using the obtained *C. burnetii IS1111* transposase sequence along with 22 reference strains (Fig. 2).

## Discussion

In Portugal, studies on coxiellosis have been developed not only in domestic animals and wildlife but also in zoo animals (Cumbassá et al. 2015; Cruz et al. 2018a; Anastácio et al. 2022; Pires et al. 2023). However, the epidemiology of *C. burnetii* remains poorly understood in the country, particularly in terms of bacterial excretion in ruminants’ milk.

Our study initially screened sheep BTM by qPCR followed by confirmation with endpoint PCR amplification and bidirectional sequencing. Analysis showed that only one positive sample was found, from a sheep farm located in Gouveia municipality in 2015 (accession no: OL310491), showing closer identity with an isolate from a goat from the Netherlands (accession no: CP013667), as shown also by the phylogenetic analysis.

As with the present study, previous studies in Europe have also used PCR methods to assess the prevalence of *C. burnetii* in BTM from sheep. In the Netherlands, investigation of BTM samples from dairy sheep farms indicated a prevalence of 0% (van den Brom et al. 2012). A survey in northern Spain focusing on ovine BTM samples, revealed a *C. burnetii* prevalence of 22% (García-Pérez et al. 2009). Curiously, in Portugal, a survey on BTM samples, conducted in the same region as the present study, found a higher prevalence of 5.1% of *C. burnetii* in sheep milk despite the higher percentage of antibody-positive in dairy sheep herds (Anastácio et al. 2016). The occurrence of *C. burnetii* detected in our study is lower than previously reported in BTM of sheep in Portugal and Spain. The present study suggests that while the prevalence of *C. burnetii* in sheep milk varies across regions, factors such as herd size and management practices likely influence its occurrence, with the current study indicating lower prevalence compared to previous reports in the region (Ryan et al. 2011; Schimmer et al. 2011; Agger et al. 2013). Additionally, differences in DNA extraction methods from milk samples may impact extraction efficiency, and variations in PCR protocols, even when targeting the same gene, could alter its sensitivity.

The samples used in this study were previously subjected to serological testing using an ELISA assay (Cruz et al. 2018b). In that prior investigation, from the 2015 sampling, eight (10.2%; 95%CI: 4.5–19.2) out of the 78 bulk tank milk samples showed IgG antibodies against *C. burnetii*, while from the 2016 sampling, 20 (25.6%; 95%CI: 16.4–36.8) out of the total 78 bulk tank milk samples tested positive. The present study revealed one positive sample from 2015 using PCR methods. Certainly, PCR results are not reliable for determining the infection status of the herd due to the variability in shedding patterns, including different shedding routes and potential intermittent shedding. The prevalence of *C. burnetii* in milk is anticipated to be higher in bovine milk than in small ruminants’ milk, given that it is the primary shedding route for cows (Guatteo et al. 2012), while in small ruminants, birth products serve as the main source of shedding (van den Brom et al. 2012). Despite the observation of *C. burnetii* excretion in sheep milk for up to 4 months postpartum (Astobiza et al. 2010), this bacterium is typically excreted in sheep milk for up to 8 days (Roest et al. 2011, 2012). Given that the calving season typically occurs between October and November and the samples were collected in January and February, this disparity in results could be explained by the fact that *C. burnetii* may no longer be excreted in milk 3 to 4 months after calving. Furthermore, the fact that we only sampled at two time points, with one year apart, further limits the possibility of detecting bacterial excretion in milk. Additionally, at the herd level, the detection of this bacteria could be affected, given that kidding events are typically grouped.Among the foods of animal origin, raw milk is regarded as the most significant source of *C. burnetii* (Panel and Ahaw 2010; Gale et al. 2015). Interestingly, *C. burnetii* exhibits spore-like stability in the environment attributed to its SCV morphotype, which is presumed to persist in milk; however, replication of this bacterium is not likely to occur in this matrix, since replication outside of the intracellular environment of host cells is not possible (Gale et al. 2015). At present, the lack of sufficient data, such as dose–response and survival in milk or milk products over time, inhibits accurately assessment of the risk of infection from consuming milk and milk products (Gale et al. 2015).

One limitation of this research is the absence of an assessment regarding the infectivity of *C. burnetii*, which could have been ascertained through in vitro isolation techniques or, less commonly, via acellular media (Shi et al. 2018). The isolation of *C. burnetii* represents a complex and laborious task, and poses a considerable risk of infection, requiring the expertise of trained professionals and a biosecurity level 3 laboratory (Sewell 1995). Therefore, the detection of *C. burnetii* in biological samples typically relies on molecular techniques like PCR (Arricau-Bouvery and Rodolakis 2005), an assay that does not enable differentiation between viable and non-viable bacteria. Another limitation of our study is the absence of bacterial quantification in the bulk tank milk samples. To accurately quantify bacteria in bulk samples using real-time PCR, amplification of a unique and specific sequence is recommended (WOAH 2018). In our study, the qPCR targeted the *IS1111* transposase, which exists in varying copy numbers depending on the isolate.

In Portugal, Q fever in humans is subject to obligatory notification, nonetheless it is reported to be largely underestimated (Palmela et al. 2012). Q fever primarily manifests as flu-like symptoms and for this reason can likely be disregarded in the diagnostic algorithm. However, it is worth noting that Q fever is re-emerging as a zoonotic disease (Arricau-Bouvery and Rodolakis 2005; Panel and Ahaw 2010). Therefore, it is advisable to consider supporting prophylactic programs that include the screening for *C. burnetii* in milk.

In summary, while our study reports a lower occurrence of *C. burnetii* in milk in central Portugal compared to other regions, caution is warranted due to variations in molecular assays and production practices. The presence of *C. burnetii* in raw milk suggests the importance for further research, particularly regarding the risk of infection from consuming milk and milk products, like artisanal cheese. Furthermore, new studies should be conducted to better understand the challenges posed by *C. burnetii* in Portugal, which will contribute to interdisciplinary efforts aimed at enhancing surveillance, prevention, and control measures against this emerging zoonotic disease.

## Data Availability

No datasets were generated or analysed during the current study.
